# Cultured rat aortic vascular smooth muscle cells do not express a functional TRPV1

**DOI:** 10.1371/journal.pone.0281191

**Published:** 2023-02-14

**Authors:** Tina Blažević, Cosmin I. Ciotu, Markus Gold-Binder, Elke H. Heiss, Michael J. M. Fischer, Verena M. Dirsch

**Affiliations:** 1 Department of Pharmaceutical Sciences, Faculty of Life Sciences, University of Vienna, Vienna, Austria; 2 Institute of Physiology, Center for Physiology and Pharmacology, Medical University of Vienna, Vienna, Austria; University of Hull, UNITED KINGDOM

## Abstract

We showed previously that capsaicin, an active compound of chili peppers, can inhibit platelet-derived growth factor-induced proliferation in primary rat vascular smooth muscle cells (VSMCs). The inhibition of BrdU incorporation by capsaicin in these cells was revoked by BCTC, which might be explained by a role of TRPV1 in VSMCs proliferation. To further pursue the hypothesis of a TRPV1-dependent effect of capsaicin, we investigated TRPV1 expression and function. Commercially available antibodies against two different TRPV1 epitopes (N-terminus and C-terminus) were rendered invalid in detecting TRPV1, as shown: i) in western blot experiments using control lysates of TRPV1-expressing (PC-12 and hTRPV1 transfected HEK293T) and TRPV1-downregulated (CRISPR/Cas gene edited A10) cells, and ii) by substantial differences in staining patterns between the applied antibodies using fluorescence confocal microscopy. The TRPV1 agonists capsaicin, resiniferatoxin, piperine and evodiamine did not increase intracellular calcium levels in primary VSMCs and in A10 cells. Using RT qPCR, we could detect a rather low TRPV1 expression in VSMCs at the mRNA level (Cp value around 30), after validating the primer pair in NGF-stimulated PC-12 cells. We conclude that rat vascular smooth muscle cells do not possess canonical TRPV1 channel activity, which could explain the observed antiproliferative effect of capsaicin.

## Introduction

Transient receptor potential cation channel subfamily V member 1 (TRPV1) is a cation channel that acts as a molecular integrator of chemical and physical pain-eliciting stimuli. It becomes activated by a variety of different ligands, but also low pH (<5.9) and noxious heat (>43°C) [1]. Structural elucidations using electron cryo-microscopy revealed that TRPV1 is a tetramer around a central ion pathway, which shows a wide extracellular opening with a short selectivity filter [2]. Each monomer contains an N-terminal domain with ankyrin repeats, 6 transmembrane helices (S1-S6), an intervening pore loop between S5 and S6, a highly conserved TRP domain and a C-terminal domain. N- and C-termini are both facing the intracellular environment. TRPV1 is permeable for monovalent cations, but even more so for bivalent cations, especially Ca^2+^ [3]. TRPV1 was originally discovered as a receptor of capsaicin, a pungent compound of chili pepper [4–6]. Soon after, many other naturally occurring compounds, e.g. resiniferatoxin [7], piperin [8], evodiamine [9] and some toxins, as well as various endogenous inflammatory mediators were found to activate TRPV1 [10]. TRPV1 was discovered at small-diameter, Aδ and C fiber, sensory nerves and the respective neurons are the common model to investigate these channels [11]. However, in the last few decades, many studies located TRPV1 in a variety of non-neuronal cells as well, e.g. urothelium epithelial cells [12], keratinocytes [13], glial cells [14] and cells of the circulatory system [15, 16].

Vascular smooth muscle cells (VSMCs) regulate the vascular tone through active contraction/relaxation in response to external stimuli. In pathologies, VSMCs lose their ability to contract and start migrating and proliferating into the intimal area. Calcium signaling plays a major role in VSMCs phenotype switching: not only does it regulate VSMCs contractility, but it also affects protein synthesis and cell cycle progression through Ca^2+^-dependent transcription factors and Ca^2+^-binding effector proteins, respectively [17]. Extracellular Ca^2+^ enters VSMCs mainly by opening of voltage-dependent L-type calcium channels, but several other channels, including TRPs, are also involved [18]. Functional TRPV1 in VSMCs was suggested by Kark et al., as capsaicin elicited vasoconstriction in isolated pressurized rat skeletal muscle arterioles, and the effect was not abolished by endothelial denudation or by the inhibition of nitric oxide synthase [15]. Only a few years later, the vasoconstrictive effect of capsaicin in arterioles of the skeletal muscle and in a subset of thermoregulatory tissues outside of the central nervous system was causally linked to VSMC-located and functional TRPV1 [19, 20]. The net effect of capsaicin depends on the concentration: lower concentrations (up to 1 μmol/L) induced a calcitonin gene-related peptide (CGRP)-dependent vasodilatation, higher concentrations induced vasoconstriction in rat meningeal vessels [21].

Capsaicin administration was described to ameliorate vascular smooth muscle foam cell formation and to attenuate atherosclerosis in ApoE knockout mice on a high-fat diet, but not in ApoE/TRPV1 double-knockout counterparts [22]. A role for TRPV1 in cell proliferation was suggested by Wang et al. as the common TRPV1 antagonist at that time–capsazepine—reduced cellular calcium levels and proliferation in hypoxia-induced human pulmonary smooth muscle cells [23]. In addition, our group reported an antiproliferative effect of capsaicin in platelet-derived growth factor (PDGF)-activated primary rat aortic VSMCs [24].

There is strong evidence supporting a functional TRPV1 expression in VSMCs of small arterioles [15, 19, 20, 25], with a recent study even describing arteriolar TRPV1 as an important player in rapid myogenic responses [26]. However, several studies have been published over the past decade with conflicting results for VSMCs from other vascular beds. Ma et al. demonstrated TRPV1 expression in aortic VSMCs from WT, but not from TRPV1 knockout mice on protein, mRNA and functional level [22]. Sand, Grant et Nandi observed immunoreactivity also in TRPV1 knockout samples, questioning the specificity of the antibody. In the same study, the authors could not reproduce functional expression of TRPV1, as capsaicin could not induce calcium influx either in aortic VSMCs or endothelial cells [27]. In 2013, Toth et al. postulated that TRPV1 expression in VSMCs might be differentially regulated in various vascular beds, as capsaicin evoked vasoconstriction in skeletal muscle arteries and in the carotid artery, but not in the femoral and mesenteric arteries or in the aorta [28]. Recently, capsaicin was shown to elicit vasorelaxation in rat aorta, but this effect could not be causally linked to TRPV1 [29]. The latter showcases that the capsaicin concentration needs to be considered and that there are limits to capsaicin specificity [30].

Capsaicin reportedly shows a plethora of effects on VSMCs, many of them considered protective in metabolic syndrome- and vascular wall-related diseases [31–33]. It would be important to know whether functional VSMC-located TRPV1 could be a potential driver. Here we report results collected from immunochemistry, functional and gene expression experiments that emerged during mode-of-action studies on capsaicin and VSMC proliferation.

## Materials and methods

### Materials

Primary rat aortic VSMCs, growth media, and cell culture supplements were purchased from Lonza (Basel, Switzerland). Fetal bovine serum for cell culture was supplied by Gibco Life Technologies (Darmstadt, Germany), and PDGF-BB was obtained from Bachem (Weilheim, Germany). Rat phaeochromocytoma cells (PC-12) cells were a kind gift from Prof. Harald Sitte (Medical University of Vienna). Horse serum and NGF were from Fisher Scientific (Vienna, Austria). The A10 cell line was purchased from LGC Standards (Teddington, UK). HEK293T cells were purchased from Merck (Darmstadt, Germany). Sprague Dawley rats (Charles River, MA, USA) were used for harvesting dorsal root ganglia. Biological material was only obtained after sacrificing the animal, which does not require explicit approval by an ethics committee under local jurisdiction. Capsaicin and BCTC were obtained from R&D Systems (Abingdon, UK) and Cayman Chemical (Ann Arbor, MI, USA), respectively. All other chemicals were purchased from Sigma-Aldrich (MO, USA).

### Cell culture

Primary VSMCs were cultivated in DMEM-F12 (1:1) supplemented with 20% fetal bovine serum, 30 μg/mL gentamicin, and 15 ng/mL amphotericin B at 37°C in an incubator with 5% CO_2_ flow in a humidified atmosphere. Passages 4 to 12 were used in experiments. Cultivation and differentiation of PC-12 cells are described in the supplementary information. A10 cells were cultivated in DMEM supplemented with 10% fetal bovine serum and 2 mM L-glutamine at 37°C in an incubator with 5% CO_2_ flow in a humidified atmosphere.

### Generating an A10 TRPV1 KO cell line using CRISPR/Cas

The A10 cell line was derived from the thoracic aorta of embryonic rat and is a commonly used model of VSMCs [34]. A10 TRPV1 KO cell line strains, H3 and G12, were generated using the CRISPR/Cas method. This part of the work was performed by the Vienna BioCenter Core Facilities (GmbH), within the group of Dr. Krzysztof Chylinski. Details are described in the supplementary information.

### SDS-PAGE and immunoblot analysis

VSMCs and A10 (WT and TRPV1 KO) were seeded at a density of 0.4 × 10^6^ cells per 6 cm dish and cultivated in growth medium for 48 h. One set of VSMCs was serum-deprived for the last 24 h of the incubation period. The same number of PC-12 cells was seeded in 10 cm dishes. Undifferentiated and differentiated PC-12 were cultivated as described in the supplementary material for 2 and 11 days, respectively. The transfection of HEK293T cells with human TRPV1 was performed using jetPEI transfection reagent (Polyplus Transfection, Illkirch, France) according to the manufacturers protocol. Afterward, cells were lysed for 20 min with an ice-cold lysis buffer (50 mM Tris-HCl pH 6.8, 500 mM NaCl, 1% (v/v) NP40, 0.5% (w/v) Na-deoxycholate, 0.1% (w/v) SDS, 0.05% (w/v) NaN_3_), supplemented with 1 mM PMSF, 1 x Complete^TM^ (Roche Applied Science), 1 mM NaF and 1 mM Na_3_VO_4_. Lysates were centrifuged at 11000 x g at 4°C for 20 min and supernatants were used for protein denaturation in 3 x SDS sample buffer for 8–10 min at 95°C. Protein concentrations were determined using Rotiquant reagent according to the manufacturer’s instructions (Carl Roth). Protein extracts (40 μg) were loaded on a 6% PAA separation gel, subjected to SDS-PAGE and immunoblot analysis. The following primary antibodies were used in this study: vanilloid R1/TRPV1 antibody (NBP1-97417, Novus Biologicals, Littleton, CO, USA) against the N-terminus of rat TRPV1; anti-VR1 antibody [BS397] against the C-terminus of mouse/rat TRPV1 (ab203103, Abcam, Cambridge, UK); and α/β-tubulin antibody (#2148, Cell Signaling Technology, Frankfurt a.M., Germany). Secondary horse-radish peroxidase-conjugated antibodies against rabbit and mouse IgG were also from Cell Signaling. All antibodies were diluted as recommended by the providing company. Proteins were visualized using enhanced chemiluminescence reagent and quantified using an LAS-3000 luminescent image analyzer (Fujifilm) with AIDA software (Raytest).

### Calcium microfluorimetry

Cells were plated on 12 mm glass coverslips. The coverslips were loaded with Fura-2 (3 μmol/L) for 30 min at 37°C and 5% CO_2_ before transfer into glass-bottom dishes in extracellular solution. After a recovery period of 10 min the dishes were mounted onto an Olympus IX73-inverted microscope and imaged using a 10x objective. Cells were continuously superfused with extracellular solution (in mmol/L): 145 NaCl, 5 KCl, 10 glucose, 10 HEPES, 1.25 CaCl_2_, and 1 MgCl_2_, buffered to pH 7.4 with NaOH and with an osmolarity of 300 mOsm. The experimental protocol was software-controlled using an 8-channel gravity-driven common-outlet system (ALA Scientific Instruments Inc, Farmingdale, NY). After a recording of baseline calcium levels, this superfusion was switched to different substances diluted in extracellular solutions. At the end of the recording, cells were exposed to 2 μmol/L ionomycin, which served as positive control. The agonists used in these experiments were capsaicin, evodiamine, resiniferatoxin and piperine. Fura-2 was alternatingly excited for 30 ms by a 340-nm LED (50 mW, operating at 100% capacity) and by a 385-nm LED (1435 mW, operating at 5% capacity) using an Omicron LEDHub (Laserage-Laserprodukte GmbH, Rodgau-Dudenhofen, Germany). Fluorescence emission was long-pass filtered at 495 nm, and pairs of images were acquired at a rate of 1 Hz with a 4.2-megapixel 16-bit CCD camera (6.5-μm pixel edge length, Prime BSI; Teledyne Photometrics, Tucson, AZ). The hardware was controlled by the μManager 1.4 plugin in ImageJ. The background intensity was subtracted before calculating the ratio between the fluorescence emitted when the dye was excited at 340 nm and at 385 nm (F340/F385 nm). The time course of this ratio was analyzed for regions of interest adapted to individual cells.

### Immunofluorescence staining

VSMCs were seeded on gelatine-coated coverslips at a density of 0.05 x 10^6^ per well in 12-well plates and grown for 24 h. Cultured cells were then labeled with 250 nmol/L MitoTracker^®^ Deep Red FM (Ex/Em 644/665 nm, Molecular Probes Inc, Eugene, OR, USA) for 30 min, washed with prewarmed medium and fixed with 4% methanol-free *p*-formaldehyde for 10 min, all in the dark. Fixed cells were permeabilized using 0.2% Triton^®^ X-100 in PBS for 10 min, washed with 1xTBS-T and blocked in 10% goat serum for 1 h at RT. Coverslips with cells were incubated with primary antibodies against the N-terminus (Novus Biologicals) and against the C-terminus of TRPV1 (Abcam), diluted as recommended by the providing company, overnight at +4°C, followed by incubation with the FITC-conjugated goat anti-rabbit IgG (H + L) or goat anti-mouse IgG secondary antibodies (Ex/Em 495/518 nm), respectively, for 1 h at RT. One sample stained with the secondary antibody only was used to examine for a potential unspecific staining (images accessible at: https://phaidra.univie.ac.at/o:1611051). After a short counterstain with 1 μg/ml Hoechst dye 34580 (Ex/Em 392/442 nm) and one washing step with PBS, coverslips were mounted on microscope slides using mounting medium, kept in the dark overnight and analyzed by using the fluorescence unit of the confocal fluorescence microscope (Leica Microsystems, Wetzlar, Germany). Samples were detected with 63x magnification, constant gain and exposure time.

### RT qPCR

A primer for rat TRPV1 (fw: TTCACCGAATGGGCCTATGG and rev: TGACGGTTAGGGGTCTCACT) was custom designed using the “NCBI” online primer design tools and oligonucleotides were synthesized by Invitrogen (USA). After the primer pair was shown to be specific and efficient in rat PC-12 cells (procedure described in the supplementary information), it was used to examine the expression of TRPV1 transcripts in VSMCs and A10 cells. Rat GAPDH (Qiagen, QT00199633) and rat 18S mRNA (Qiagen, QT02589300) were used as the control housekeeping genes. To quantify mRNA levels, 0.6 x 10^6^ cells were seeded in 6 cm dishes and grown for 48 h while, in the case of VSMC, one set of cells was serum-deprived for the last 24 h of the cultivation. Total RNA was isolated with peqGOLD total RNA Kit (Peqlab, Germany) according to the manufacturer’s protocol (for the isolation of RNA from DRGs, see supplementary material). cDNA was transcribed from 1 μg of total RNA using the High Capacity cDNA Reverse Transcriptase Kit from Applied Biosciences (Fisher Scientific, Vienna, Austria) and assayed in triplicate by RT qPCR on a LightCycler^TM^ 480 (Roche Diagnostics, Switzerland) using Luna Universal qPCR Master Mix (New England Biolabs, Ipswich, MA, USA), under conditions recommended by the provider. Relative quantification of the copy number for TRPV1 in CRISPR/Cas edited A10 cells was performed using the 2^-ΔΔC^_T_ method.

### Statistics

For BrdU incorporation and RT qPCR experiments statistical analyses were performed using Prism version 9 (GraphPad Software, La Jolla, CA, USA) for a minimum of 3 independent biological experiments. Data are expressed as means ± SD. Statistical significance (P < 0.05) was determined by one-way ANOVA (Tukey post-hoc test).

## Results

### Examining protein expression of TRPV1 and its role in the antiproliferative action of capsaicin in aortic VSMCs

At first, we aimed to explore whether primary rat aortic VSMCs express TRPV1 and whether the antiproliferative effect of capsaicin could be overruled by a specific TRPV1 antagonist. To examine the protein expression of TRPV1 by western blot analysis, we used two different commercially available antibodies: a rabbit polyclonal antibody against the N-terminus and a mouse monoclonal antibody against the C-terminal end of TRPV1. PC-12 were reported to express functional TRPV1 and to show increased neuritogenesis upon nerve growth factor (NGF) stimulation [35–37]. We therefore loaded whole cell lysates of both unstimulated and stimulated PC-12 cells on the same SDS-PAGE gel with VSMCs lysates, as a positive control. PC-12 cells were stimulated by NGF as described in the supplementary information. To ensure a negative control for the above-mentioned antibodies, we resorted to the CRISPR/Cas technology to create a TRPV1 knockout in the A10 vascular smooth muscle cell line [34]. A cell line was used, because the CRISPR/Cas technology requires long cultivations and many passages to obtain knockout clones, which is not possible with primary cells. Successful gene editing was confirmed by genotyping, as described in the supplementary methods (detailed information: https://phaidra.univie.ac.at/o:1611051**)**, for two different A10 TRPV1 knockout clones, designated as G12 and H3. The CRISPR/Cas-induced indels in these two clones were within exon 2 (and additionally in intron 1 in the H3 strain) and are likely to result in premature translation termination/transcript degradation. We therefore did not expect to detect the TRPV1 protein with antibodies against either the N-terminus or C-terminus in any of the A10 TRPV1 knockout cells. Capsaicin inhibited proliferation under conditions of a minute serum amount. Serum deprivation can result in significant perturbations of protein expression profiles in cultured cells [38]. Therefore, lysates of both growing and quiescent VSMCs were loaded on the gel not to overlook changes in TRPV1 expression potentially induced by serum-deprivation.

**Fig 1:**
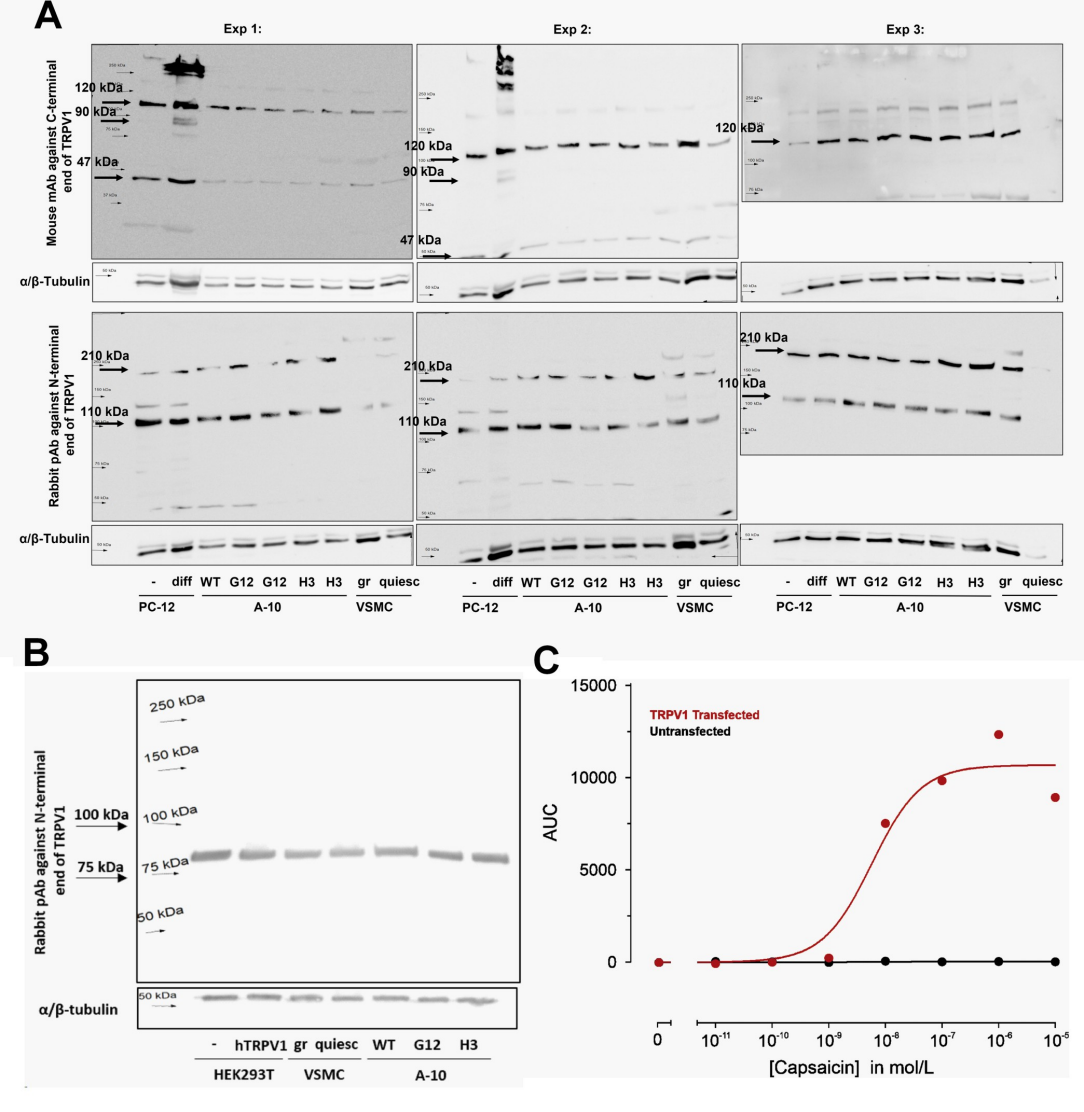
Unspecific immunoreactivity of antibodies against TRPV1. Western blot detections of TRPV1 protein in undifferentiated (-) and differentiated (diff) PC-12 cells (A), wild-type (WT) and TRPV1 KO (G12, H3) A10 cells (A and B), in growing (gr) and quiescent (quiesc) rat primary VSMCs (A and B), and in TRPV1 transfected HEK293T cells (B) using two different antibodies against TRPV1: mouse monoclonal antibody (mAb) against the C-terminus (A, upper panel) and rabbit polyclonal antibody (pAb) against N-terminus (A, lower panel and B), α/β-tubulin was used as a loading control. In each experiments cells of different passages were used. The membrane in experiment 3 was cut in the attempt to reduce unspecific binding by exposing only the part where TRPV1 was expected to bind the primary antibody. (b) One representative blot out of 4 independent western blot experiments with similar results is shown. (C) Capsaicin concentration-response curves measured by calcium microfluorimetry. The response to capsaicin was quantified by the area under the curve (AUC) and fitted by a sigmoidal function. HEK293T cells transfected with human TRPV1 (red) respond to capsaicin, in contrast to untransfected HEK293T cells (black).

The expected size of the detected TRPV1 protein was around 100–110 kDa. Strong bands at 110 and 120 kDa were detected in all loaded samples with antibodies against both the N-terminus and C-terminus of TRPV1, respectively (Fig 1A). We expected a more intensive band at the respective size in NGF-stimulated and differentiated PC-12 cells, compared to their unstimulated counterpart. However, the differences in intensity that could be observed (Fig 1A) were due to differences in protein loading. Only in two out of three membrane detections using the monoclonal antibody against the C-terminus, we observed one additional double-band in NGF-stimulated PC-12 that corresponds to a protein of ca. 90 kDa size. This band was not present in either unstimulated PC-12 or in samples from A10 and primary VSMCs (Fig 1A). In addition, a prominent immunoreactive band at 110/120 kDa size was detected in A10 WT, as well as in TRPV1 KO cells (Fig 1A). We therefore do not believe that these two commercially available antibodies detect TRPV1 at 110/120 kDa. Other strong bands were visible in all samples: at ca. 47 and 210 kDa in the case of the antibody against the C-terminus and against the N-terminal end, respectively (Fig 1A), further corroborating the unspecific binding.

Moreover, when using human TRPV1 overexpressing HEK293T cells as a positive control in western blot studies with our vascular smooth muscle cell models, there was no difference in intensities of “TRPV1 bands” between untransfected and transfected cells (Fig 1B). The reason for this could not have been an unsuccessful transfection, as only TRPV1-transfected HEK293T cells took up calcium after exposure to capsaicin. The effect was concentration-dependent between 10 nmol/L and 10 μmol/L capsaicin (Fig 1C). Of note, by using the overexpression approach, we could validate only the polyclonal antibody against the N-terminal end of the TRPV1 (Novus Biologicals), as it was reported by the company to react to both rat and human protein. Different appearances of blots in Fig 1A (lower panel) and 1B could only be explained by different batches of the antibody, which indicates the product marketed by Novus Biologicals shows inconsistent results.

**Fig 2:**
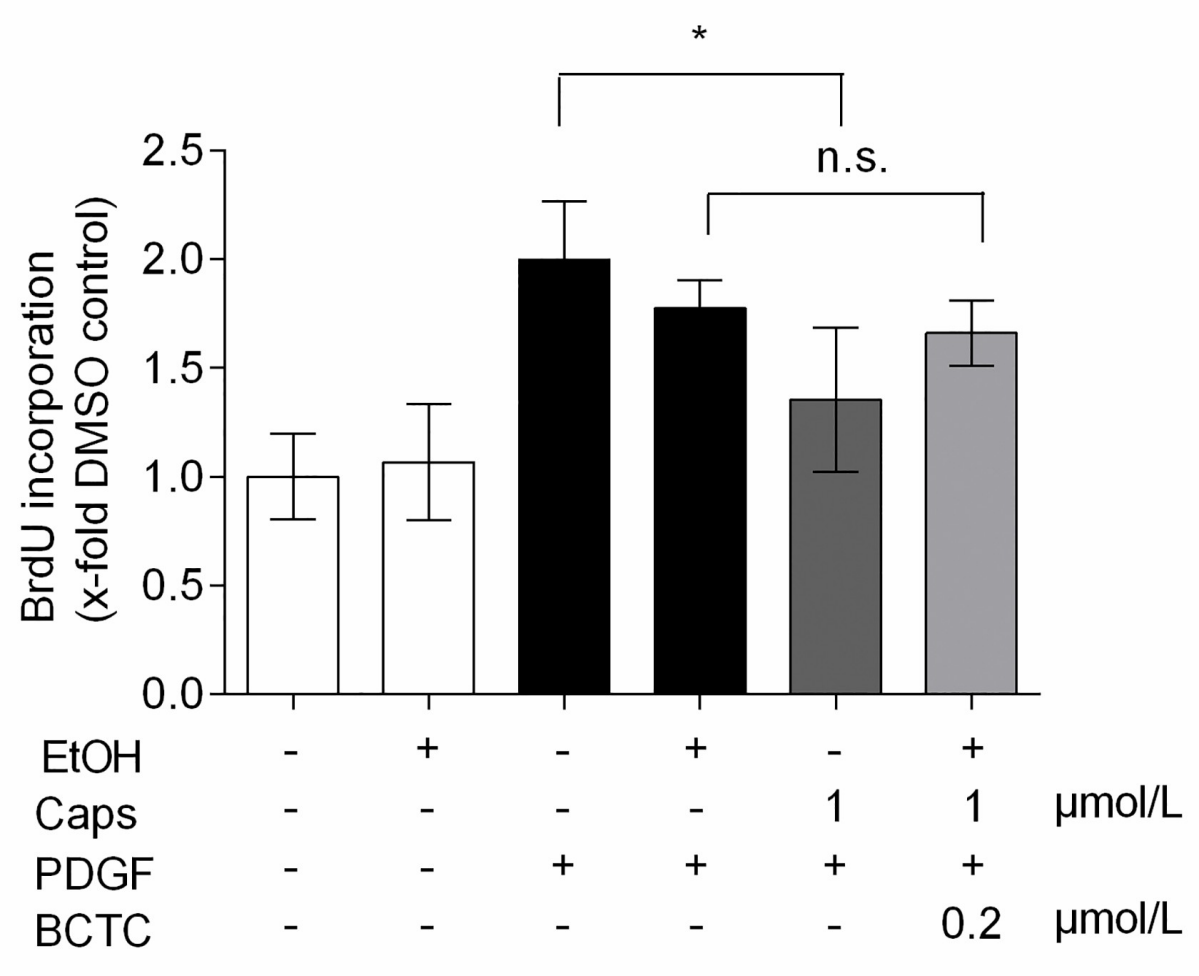
The antiproliferative effect of capsaicin in VSMCs diminishes in the presence of BCTC. Quiescent VSMC were pretreated with vehicle controls (0.1% DMSO alone, or in combination with 0.1% ethanol, EtOH), 1 μmol/L capsaicin (Caps) alone or in combination with 200 nmol/L BCTC for 30 min and incubated for 24 h with 20 ng/mL PDGF-BB. The graph shows the mean values ± SD of incorporated BrdU relative to DMSO vehicle control out of 3 independent experiments, each performed in triplicates (* p < 0.05; n.s. not significant; ANOVA, Tukey post-hoc test).

Co-treatment of VSMC with capsaicin and the potent TRPV1 antagonist BCTC abolished the significant reduction of PDGF-induced BrdU incorporation, and thus *de novo* DNA synthesis, seen with capsaicine alone (Fig 2). In TRPV1-transfected HEK293T cells, the capsaicin-induced calcium influx was antagonized by BCTC within the concentration range used in the co-treatment experiment (S1 Fig), demonstrating the on-target effect of this antagonist. Checking protein expression by western blot did not allow any reliable conclusion on TRPV1 expression in rat VSMCs. However, a co-treatment study suggested a potential role of TRPV1 in the observed activity of capsaicin. We therefore decided to further examine the debated issue of expression and functionality of TRPV1 in primary rat aortic VSMCs.

### Lack of functional expression of TRPV1 in rat aortic VSMCs

**Fig 3:**
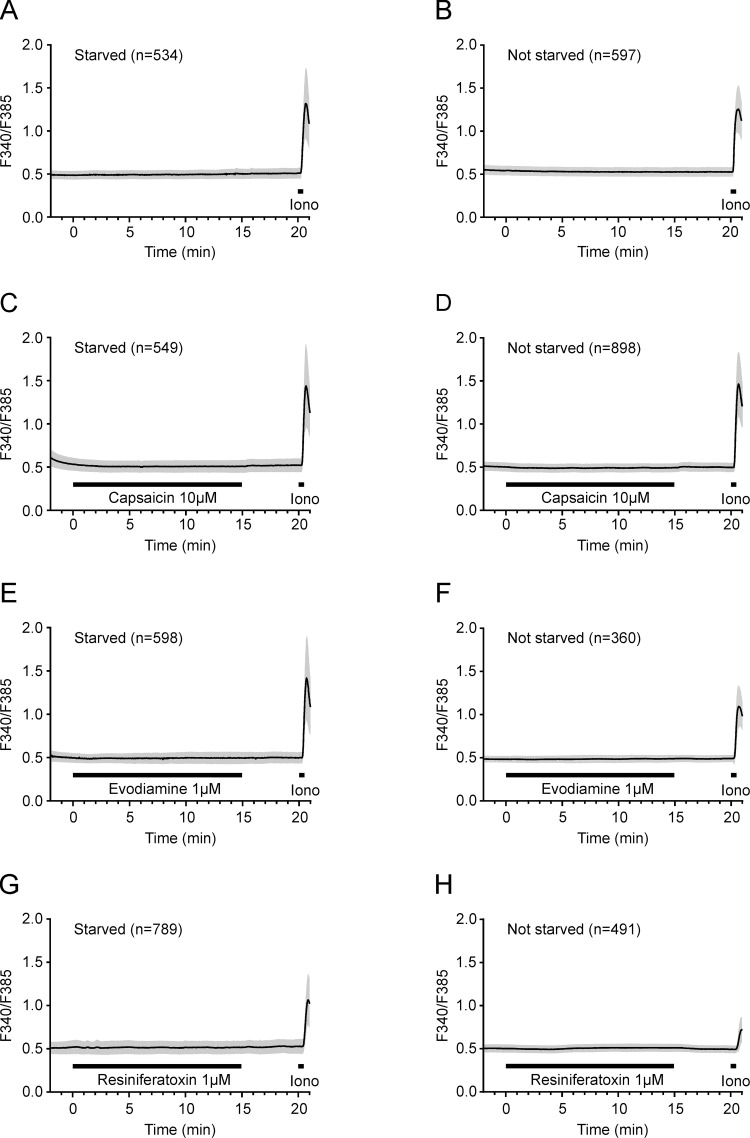
TRPV1 agonists do not elicit Ca^2+^ influx in rat aortic VSMCs. Intracellular calcium time courses in primary VSMCs under serum starvation (A, C, E, G) or with normal FBS supplementation (B, D, F, H) and prolonged exposure to TRPV1 agonists are presented as mean ± SD. Cells were exposed to extracellular solution (A, B), capsaicin 10 μmol/L (C, D), evodiamine 1 μmol/L (E, F) and resiniferatoxin 1 μmol/L (G, H) for 15 minutes, and calcium influx was measured as described in Materials and Methods. A control application of ionomycin 2 μmol/L at the end of each protocol served as a technical positive control. The number of cells is indicated by “n”.

To examine the functionality of TRPV1, intracellular calcium in both growing and quiescent VSMCs was monitored using the Fura-2 AM dye during 15-minute exposures to different known TRPV1 agonists: capsaicin, resiniferatoxin, piperine and evodiamine. Although a strong and immediate effect could be detected upon exposure to the positive control, ionomycin, all tested TRPV1 agonists failed to induce a relevant increase in intracellular calcium levels in VSMCs, irrespective to the presence or absence of serum (Fig 3). Piperine has excitation and emission wavelengths of 339 nm and 450 nm [39], which substantially interfered with the working range of the Fura-2 dye, and precluded recordings (https://phaidra.univie.ac.at/o:1611051). These results show that VSMCs do not express functional TRPV1. Additionally, A10 cells were challenged with the same stimuli but did not exhibit any change in intracellular calcium (S2 Fig).

### Immunostaining and subcellular localization of TRPV1 in aortic VSMCs

**Fig 4:**
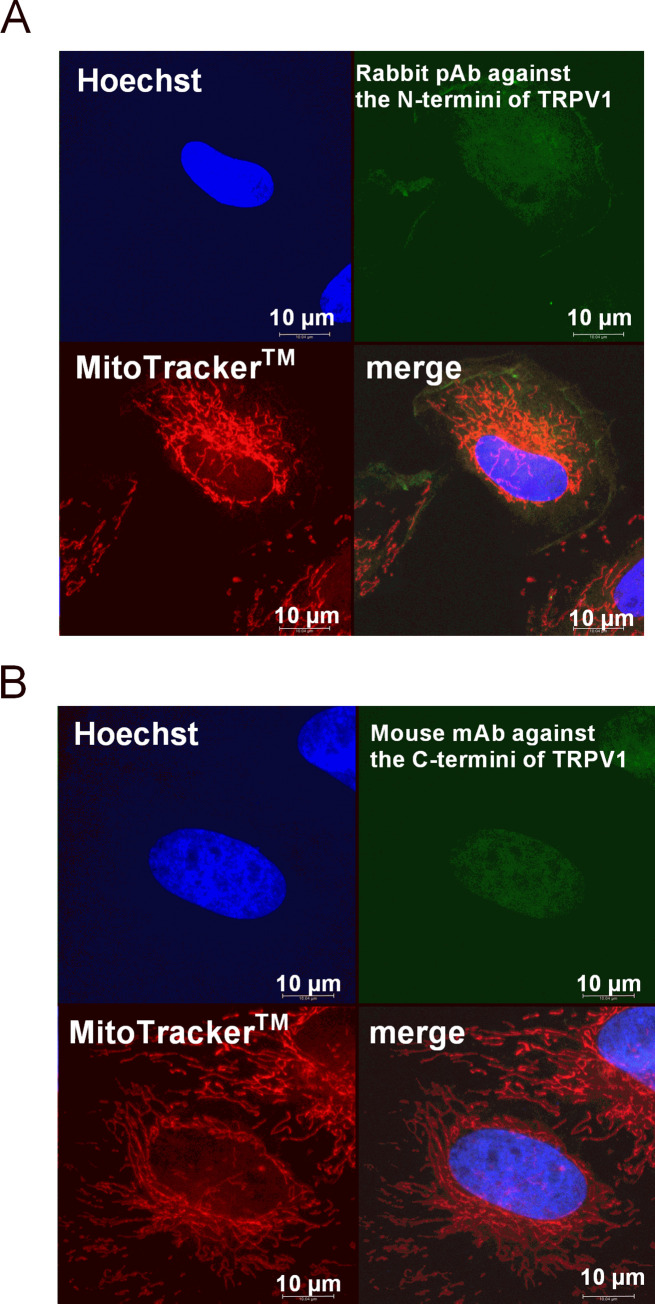
TRPV1 staining in mitochondria-labeled VSMC. Confocal microscopy images of triple-stained rat VSMCs (objective magnification 63x): Hoechst (blue) for nucleus, FITC-conjugated secondary antibody (green) bound to the (A) polyclonal antibody against the TRPV1 N-terminus or (B) monoclonal antibody against the TRPV1 C-terminus, and MitoTracker^®^ DeepRed FM (red) for mitochondria.

Despite being described by their respective companies as applicable for both western blot and immunofluorescence, above-mentioned antibodies might bind better to conformational, rather than linear epitopes. We therefore decided to explore the cellular localization of TRPV1 in fixed VSMC by immunostaining. Although originally considered to be confined within the plasma membrane, evidence for the existence of TRPV1 channels also in organellar membranes has been accumulating over the past two decades [40–42]. Whereas TRPV1 located on the ER operates by increasing the cytoplasmic Ca^2+^ concentration [43], mitochondrial TRPV1 was reported to facilitate Ca^2+^ transport from the cytoplasm into the mitochondrial matrix [44]. Assuming a capsaicin sensitivity of TRPV1 like at the plasma membrane, functional expression of TRPV1 in ER or within the inner mitochondrial membrane would most likely also have been detected in our calcium microfluorimetry experiment. Nevertheless, we double-stained our VSMCs with antibodies against the TRPV1 and with the mitochondrial dye MitoTracker® Deep Red. Confocal fluorescence microscopy images showed quite different staining patterns between the two commercially available antibodies: signals from the TRPV1 antibody against the N-termini could not be localized to a specific cellular compartment, e.g. membrane (Fig 4A), whereas a particularly strong staining was observed in the nucleus when using the antibody against C-termini (Fig 4B). Of note, the latter was not visible in serum-starved VSMCs (https://phaidra.univie.ac.at/o:1611051). The signal from the TRPV1 antibody and mitochondrial staining did not overlap in maximal projections. We believe the staining pattern is very likely a result of unspecific immunoreactivity of both antibodies, which seems to be true also for non-denatured (native) samples.

### Gene expression of TRPV1 in VSMCs and in A10 cells

Finally, we resorted to RT qPCR to obtain information on TRPV1 transcript expression in VSMCs. Custom designed primers for rat TRPV1 were tested for amplification efficiency and specificity in both differentiated and undifferentiated PC-12 cells. Only in PC-12 cells that underwent an 8-day NGF differentiation (S3A Fig), one primer with an amplification factor of ~2, a 100% amplification efficiency (https://phaidra.univie.ac.at/o:1611051), produced a single amplified product in the melting curve (S3B Fig). These results suggest that only differentiated PC-12 cells express the TRPV1 transcript, but the expression level is at the edge of detection with an undiluted Cp of ~29 compared to unspecific amplification with a Cp of ~30.

**Fig 5:**
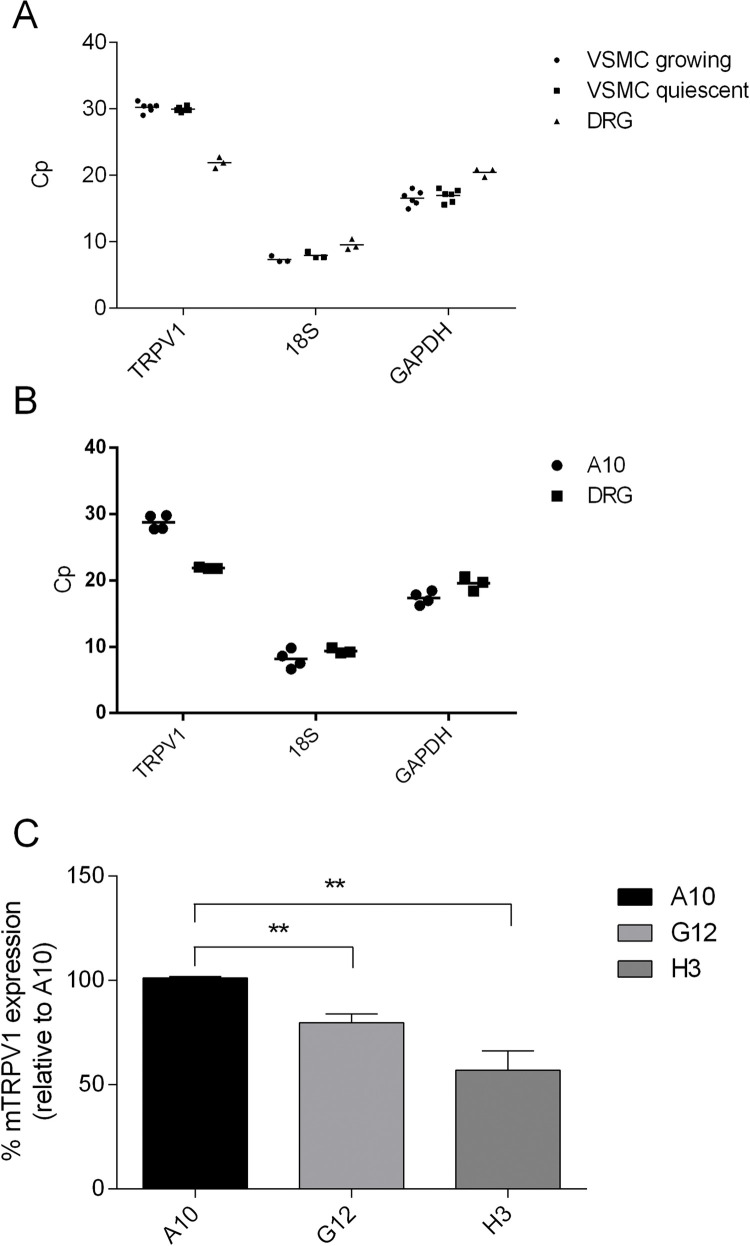
Expression of TRPV1 mRNA in cultured primary VSMC and in CRISPR/Cas-edited A10 cell line. TRPV1, GAPDH and 18S mRNA expression levels in growing and quiescent VSMCs (A), and in A10 cell line (B) determined by RT qPCR; (A and B) the graphs show Cp values from each independent biological experiment. DRG was used as a positive control. (C) Relative TRPV1 mRNA expression in CRISPR/Cas-edited A10 cells, compared to their normal counterpart; graph shows mean ± SD in the form of percentage relative to the amount of TRPV1 transcript in normal A10 cells of 4 independent biological experiments, each performed in triplicate (** P < 0.01; one-way ANOVA, Tukey post-hoc test). GAPDH was used as a housekeeping gene control.

The tested primer was then used to examine TRPV1 mRNA expression in both cultured and serum-deprived VSMCs. cDNA from rat dorsal root ganglion (DRG) was used as a positive control. In cDNA samples derived from VSMCs, the amplification signal of TRPV1 appeared around the 30th cycle, in both presence and absence of serum (Fig 5A). This again indicates a rather small amount of the transcript. The Cp value for TRPV1 in DRG was around 22, which validated the method by observing the high and well-known TRPV1 expression levels.

To make sure that the observed immunoreactivity in CRISPR/Cas edited A10 cells (Fig 1A and 1B) was not due to an unsuccessful knockout, we examined whether there are differences in TRPV1 mRNA expression between naive A10 and TRPV1 knockout strains: G12 and H3. The Cp value for the TRPV1 amplicon appeared around the 29th cycle in A10 cells, indicating a rather small amount of TRPV1 expression in these cells as well (Fig 5B). The TRPV1 amplicon was detected also in the TRPV1 knockout strains G12 and H3. However, compared to their normal counterpart, the levels were significantly reduced in both strains (Fig 5C). A somewhat stronger reduction in TRPV1 mRNA could be observed in the H3 strain (around 40%), probably due to deletion of over 500 nucleotides compared to +1/-1/-7 indels in G12 strain (as confirmed by single allele genotyping). Although CRISPR/Cas gene editing did not produce a complete TRPV1 knockout in A10 cells, it led to a reduced TRPV1 mRNA expression, either due to reduced gene transcription or production of a less stable truncated product.

## Discussion

We aimed to examine expression and functionality of TRPV1 in rat VSMCs to appreciate its potential role in the observed antiproliferative effect of capsaicin. Based on the performed immunoblot and immunocytochemistry data we could not unambiguously decide on (non)expression of TRPV1 in rat VSMCs due to the poor performance of used antibodies. qPCR indicated a minimal abundance of *trpv1* transcripts, which, however, was not reflected in functional tests, where TRPV1 ligands did not increase intracellular calcium. Thus, although BCTC was able to overcome capsaicin-mediated growth arrest in VSMCs, canonical TRPV1 function is unlikely the responsible target.

In western blot experiments, we used both positive (unstimulated vs. NGF-stimulated PC-12 cells and untransfected vs. TRPV1 transfected HEK293T cells) and negative (TRPV1 knockout vs wild-type A10 cells) controls to validate two commercially available antibodies. Our results reveal that the immunoreactive bands near the expected protein size (90–120 kDa) using both antibodies do not detect TRPV1, because: i) the antibody against the C-terminal end detected a strong band somewhat higher (at ca. 120 kDa) than the antibody against the N-terminus (at ca. 90 and 110 kDa) consistently in all three experiments and in all samples and ii) the differential immunoreactivity between unstimulated and NGF-stimulated PC-12 cells, normal and TRPV1 overexpressing HEK293T cells, as well as between TRPV1 knockout and wild-type A10 cells was missing. The latter could be expected given the observed differences in mRNA levels. The introduction of insertion-deletions by non-homologous end-joining might also result in the production of novel proteins due to alternative translation initiation and the presence of pseudo-mRNAs (as indicated by our qPCR results) and these proteins might contain the same epitope as a target protein. However, we could not observe any additional immunoreactive bands in western blot when comparing our CRISPR-Cas9-genome edited TRPV1 KO A10 cells with their parental counterpart. We conclude that the antibodies used in our study are unreliable, misleading and unspecific, potentially excepting the monoclonal antibody against the C-terminus: apart from showing several immunoreactive bands, this antibody could detect a weak double-band at ca. 90 kDa in NGF-stimulated PC-12 cells that was missing in all other samples, which could be TRPV1.

Tóth et al. showed that TRPV1 is expressed in rat aorta (among other vascular beds) by also employing antibodies against the N-terminus and the C-terminus in western blot analysis. However, they validated their antibodies by using blocking peptides in dorsal root ganglia, and not by a genetic approach [28]. Furthermore, using the same antibody as Tóth et al. and antibodies from two other companies, Sand et al. detected a band in aortic lysates from both WT and TRPV1 knockout mice [27]. This shows that also antibodies used in other studies do not detect TRPV1 at around 100 kDa size. Additional experiments on the presence of a functional channel are indispensable, not just in VSMCs, but in other cell types and tissues as well. Results presented in this study are, admittedly, limited to only cultured aortic VSMCs and whether TRPV1 is expressed in VSMC from native aortic tissue remains controversial. However, recent findings by Phan et al. in transgenic TRPV1 mice strongly indicate the lack of TRPV1 also in native VSMC of large vessels, like aorta [25]. Whether capsaicin can inhibit VSMC proliferation *in vivo* and whether this effect would be masked or further potentiated in the presence of TRPV1 in VSMC from small arterioles [19, 20, 25] warrants further investigation.

Our calcium imaging results are in contrast with the findings of Gao et al., who showed an increase in calcium influx in primary rat aortic vascular smooth muscle cells upon exposure to 1 μmol/L capsaicin [45]. This might be due to differences in fluorescent dyes that were used and possibly, in exposure times to capsaicin. However, we could not detect an increase in intracellular calcium in the presence of four different TRPV1 agonists, including the highly potent resiniferatoxin, in our cell model. Conflicting calcium imaging results were also obtained upon capsaicin treatment of VSMCs from aortas of C57BL/6J mice. Whereas Ma et al. showed a concentration-dependent increase in capsaicin-mediated calcium influx blocked by TRPV1 antagonists [22], Sand et al. obtained negative results in VSMCs and in endothelial cells using the same capsaicin concentration [27]. By using Fura-2 AM dye and the Mn^2+^ quenching approach in porcine coronary arteries, Bratz et al. localized the functional TRPV1 expression to endothelial cells [46]. We also could not show a functional TRPV1 expression in the aortic A10 cell line by calcium microfluorimetry. Taken together, we argue that aortic VSMCs do not express a functional TRPV1.

Most of the positive TRPV1 immunostaining results in VSMCs were obtained from vascular tissue samples where TRPV1 co-localized with VSMCs-specific markers [15, 19, 28, 32]. Apart from Cavanaugh et al. who localized TRPV1 within arteriolar VSMCs [19], none of the other studies validated the staining patterns by genetic approaches. Only a few studies dealt with the intracellular localization of TRPV1 in VSMCs, albeit with conflicting results and interpretations. Partly in line with our observations, uniform nuclear and cytoplasmic TRPV1 staining was observed in VSMCs of mice [22] and rat [29] aorta as well as of porcine coronary arteries [46]. However, the authors of the latter study interpreted the staining pattern to be due to unspecific antibody binding. The antibody used for staining TRPV1 in VSMCs from mice and rat aorta was validated in VSMC lysates of TRPV1 knockout mice [22], but the results did not show loading controls for WT and KO samples [22, 32]. When Gao et al. used a CuS-nanoparticle conjugated TRPV1 antibody in VSMCs of rat aorta, they observed a staining pattern limited only to the plasma membrane [45], which is contrary to our observations. Of note, in the control treatment, non-conjugated CuS-nanoparticles entered VSMCs and showed a uniform intracellular staining pattern [45], possibly due to smaller particle size. To the best of our knowledge, there is no report on the validation for the antibody used by Gao et al. by knockout approaches. We argue that the conflicting immunostaining patterns are a result of unspecific and poor antibodies against TRPV1 available on the market, as reflected in our confocal microscopy images.

We were able to detect TRPV1 transcripts in our primary aortic VSMCs and in A10 cell line by employing a primer that was tested beforehand for its specificity and amplification efficiency in unstimulated vs. NGF-stimulated PC-12 cells. TRPV1 transcripts, despite of lacking functional channels, were also found previously by others in rat and mice aortic lysates [27, 28]. The possible reason for this might be that aortic VSMCs maintain a pool of TRPV1 transcripts ready for protein synthesis in response to extracellular stimuli. Another explanation might be that VSMCs express TRPV1 splice variants that either lack the channel function [47] or become activated by means other than ligand binding, e.g., cell shrinkage and temperature >37°C [48, 49].

BCTC was described to inhibit cell proliferation at 10 μmol/L by increasing the p38 and JNK phosphorylation in prostate cancer cells [50]. We, too, could observe a reduction in PDGF-induced *de novo* DNA synthesis in VSMC at 5 μmol/L BCTC (https://phaidra.univie.ac.at/o:1611051). Although used at concentration that does not affect cell proliferation in our co-treatment experiment, BCTC might have prevented a recently described targeting of the MKK3-p38 axis by capsaicin [51, 52] to exhibit the antagonizing effect on PDGF-induced VSMC proliferation.

Capsaicin inhibited proliferation in primary rat VSMCs at concentrations that are comparable to those used to elicit vasoconstriction and calcium influx in VSMCs in other studies [15, 19], but about 100–1000 fold higher than concentrations shown to be effective in settings involving the overexpressed TRPV1 [3, 53]. Effective capsaicin concentrations for channel activation might depend on a specific cellular environment. However, based on our results on TRPV1 expression and functionality and by comparing them to other studies, we argue for a TRPV1-independent mode-of-action of capsaicin in aortic VSMCs.

## Supporting information

S1 Fig A specific TRPV1 antagonist, BCTC, overrides the effects of capsaicin in TRPV1-overexpressing HEK293T cells. Dose-response curve of the TRPV1 antagonist BCTC against capsaicin in human TRPV1-transfected HEK293T cells. Left panel: time courses of normalized fluorescence displayed as mean of two individual runs (range in grey) recorded with a FlexStation3. BCTC (1 to 1000 nmol/L) was added automatically at 20 s, capsaicin 0.5 μmol/L at 60 s. Right panel: dose response curve, given by area under the curve of fluorescence intensity over the capsaicin 0.5 μmol/L exposure. Two independent runs, represented as mean with range, were fitted with a sigmoidal function.
(TIF)

S2 Fig Expression of functional TRPV1 in A10 cells. Intracellular calcium time courses in A10 cells under prolonged exposure to TRPV1 agonists are presented as mean ± SD. Cells were exposed to extracellular solution (A), capsaicin 10 μmol/L (B), evodiamine 1 μmol/L (C) and resiniferatoxin 1 μmol/L (D) for 15 min. A control application of ionomycin 2 μmol/L at the end of each protocol served as a positive technical control. The number of cells is indicated by “n”.
(TIF)

S3 Fig Custom-designed primer for TRPV1 mRNA shows the best amplification specificity in differentiated PC-12 cells. (A) Light microscopy images (200x) of PC-12 cell differentiation into tumor glial cells. Pictures were taken every 2 to 3 days during a 10-day period of supplementation with 100 ng/ml NGF. (B) Testing of the custom-designed primer (fw: TTCACCGAATGGGCCTATGG and rev: TGACGGTTAGGGGTCTCACT) for rat TRPV1 mRNA in tumor glial cells: melting peaks of the amplified PCR product from serially diluted cDNA samples are shown.
(TIF)

S1 File Supplementary methods. (DOCX)

S1 Raw images (PDF)
