# Supplementary figures and images for: Cultured rat aortic vascular smooth muscle cells do not express a functional TRPV1
Source: PLoS One. 2023 Feb 14;18(2):e0281191. doi: 10.1371/journal.pone.0281191 (PMC9928102; doi:10.1371/journal.pone.0281191)

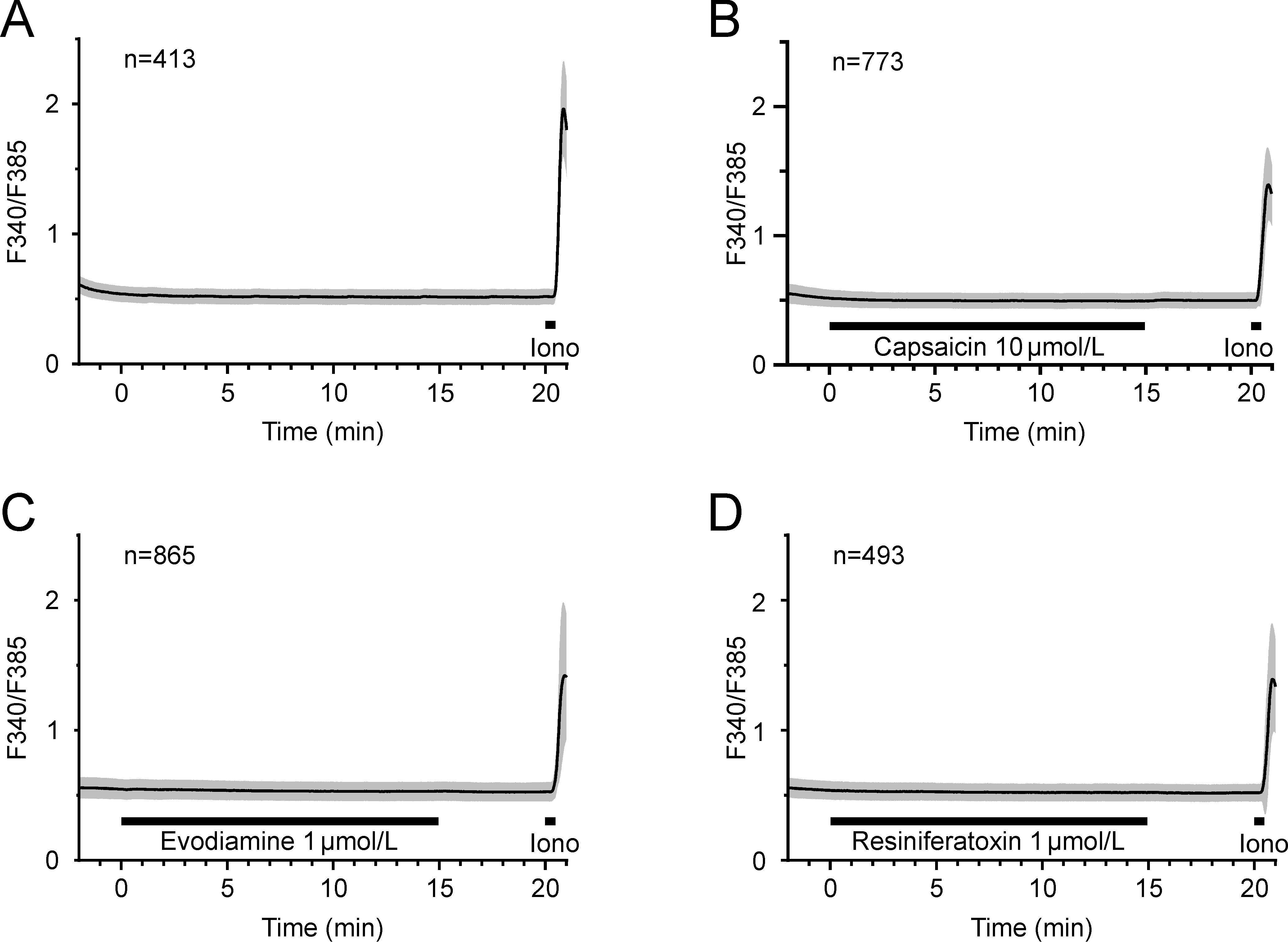

Supplement: S2 Fig — Intracellular calcium time courses in A10 cells under prolonged exposure to TRPV1 agonists are presented as mean ± SD. Cells were exposed to extracellular solution (A), capsaicin 10 μmol/L (B), evodiamine 1 μmol/L (C) and resiniferatoxin 1 μmol/L (D) for 15 min. A control application of ionomycin 2 μmol/L at the end of each protocol served as a positive technical control. The number of cells is indicated by “n”. (TIF) [file pone.0281191.s002.tif]
