# Supplementary material for: Cultured rat aortic vascular smooth muscle cells do not express a functional TRPV1
Source: PLoS One. 2023 Feb 14;18(2):e0281191. doi: 10.1371/journal.pone.0281191 (PMC9928102; doi:10.1371/journal.pone.0281191)
Supplement: S1 File — (DOCX) [file pone.0281191.s004.docx]

**Supplementary methods**

***Cultivation and differentiation of PC-12 cells***

PC-12 cells were cultivated in RPMI 1640 medium supplemented with 10% horse and 5% fetal bovine serum, and with 2 mmol/L L-Glutamine at 37°C in an incubator with 5% CO2 flow in a humidified atmosphere. For the differentiation into tumor glial cells, 0.4 x 10^6^ PC-12 cells were seeded into a collagen I rat tail-coated 10 cm dish and grown o/n in growth medium. On the next day, the differentiation was initiated by replenishing PC-12 cells with RPMI 1640 medium supplemented with 5% horse serum, 2 mmol/L L-Glutamine and 100 ng/mL of nerve growth factor (NGF) every 2 to 3 days, over a period of 8 to 12 days.

***Generating A10 TRPV1 KO cell line using CRISPR/Cas***

0.5 x 10^6^ A10 cells were electroporated with 12 µg of gRNAs targeting exons 1 (GGAGTCGTACCCGGCTTTTT, „g1244“) and 2 (CGAGGCTCTATGATCGCAGG, „g1245“) pre-mixed with 5 µg Cas9 protein (2×NLS) in Cas9 buffer (20 mmol/L HEPES pH 7.5, 150 mmol/L KCl, 0.5 mmol/L DTT, 0.1 mmol/L EDTA). Electroporations were performed using a Neon Transfection System with 100 µl Neon Pipette Tips using determined electroporation protocol of 1350 V pulse; 30 ms pulse width, 1 pulse (optimized in house by electroporation of GFP-encoding plasmid with 4 different programs). Sequences were cloned into an in house template vector p31 containing T7 promoter followed by BbsI cloning sites, optimized gRNA scaffold and DraI restriction site used for template linearization. Electroporated cells were cultured in DMEM supplemented with 10 % FCS and L-Gln. Normocin (antibiotic/antimycotic; Invivogen) was added after approximately 2 hours. 24 hours after electroporation, part of batch culture was collected for genotyping to confirm editing. Genotyping was done by PCR with following pairs of primers: „CR2896“ TTGTGGTCCCCAAGATGGAC and „CR2897“ GGGATACTTGTGTCCCCGAC for g1244 (Exon1, Expected size: 894bp) and „CR2898“ ACACTCTTGTGACCTCGCTG and „CR2899“ CTGTAGCTGTACCAGGCCA for g1245 (Exon2, Expected size: 930bp). PCR products were column purified (GeneJET Gel Extraction Kit) and send for sequencing with primer CR2898 for g1245 and CR2896 for g1244. g1245 showed the highest editing. Single clones of A10 cells electroporated with g1244 and g1245 were prepared in 96 well plates by serial dilution of culture calculated to 0.5 cells per well. Clones were cultured in medium supplemented with 50% conditioned medium (1 day old medium collected from actively growing cells) for 10 weeks with weekly media exchange. Confluent clones were collected for PCR genotyping as described above. TIDE was used to determine genotype of each clone. Large deletions resulting from g1244 and g1245 simultaneous cleavage were not detected, as examined using outside PCR. The clone IIH3 +1/-1/-536 (designated as H3) was analysed as followed: the genotyping PCR product was cloned into an in house blind cut template vector and sequenced. Single allele sequencings showed +1, -1 and additionally a large -536 deletion with an insertion of 2 nucleotides. All the cloned alleles have a -2 deletion within an intron that was as well detected in the WT cells pointing to cell line specific polymorphism. The deletion of -536 nucleotides spans between the middle of intron 1 to exon 2, deleting 65 nucleotides from exon 2, destroying exon intron junction resulting in a possible exon skipping and frame shift. Second clone IIG12 -7/-1/+1 (designated as G12) was analysed in a same way as clone IIH3. No disturbance or editing were detected at g1244. Both TIDE analysis and cloning with subsequent single alleles sequencing showed consistent frameshifts resulting from -7, -1 and +1 indels. The genotyping PCR product was cloned into an in house blind cut template vector and sequenced. Single allele sequenced shows -7, -1 and +1 genotype. A report with detailed information on CRISPR/Cas editing of A10 cell line is to be found at: https://phaidra.univie.ac.at/o:1611051.

***Proliferation assay***

VSMC were seeded at a density of 0.01 x 10^6^ cells per well in a 96-well plate, grown for 24 h and subjected to serum deprivation for the next 24 h. After a 30 min pretreatment with compounds or vehicle controls, cells were stimulated with 20 ng/ml PDGF-BB for 24 h, and labeled with 10 µmol/L bromo-deoxyuridin (BrdU) for the last 2 h of the experiment. BrdU incorporation was determined using the Cell Proliferation ELISA, BrdU (chemiluminescent) kit from Roche Diagnostics (Mannheim, Germany) as instructed by the kit manual.

***Testing the custom synthesized primers in PC-12 cells***

Custom designed primers (table S1) for rat TRPV1 were tested in undifferentiated and differentiated PC-12 cells for its amplification efficiency and specificity. In two 10 cm dishes, 0.4 x 10^6^ PC-12 cells were seeded in 10 mL growth medium. On the next day, cells were replenished with serum-reduced medium in the absence (undifferentiated PC-12 cells) or presence of 100 ng/mL NGF (differentiated PC-12 cells). These media were changed every 2-3 days over an eight-day period. Cells were then lysed, RNA extracted and cDNA was synthesized as described in the main manuscript. Water-, reverse transcriptase positive- and negative- samples, as well as following serial dilutions of cDNAs: 1:1, 1:25, 1:50, 1:100, 1:250, 1:500 and 1:1000, were subjected to RT qPCR reaction with 0.5 µmol/L of the primer set (oligonucleotide sequences are given in the main manuscript), as described in the main manuscript. The primer 1 yielded a single melting curve product, had amplification factor and primer efficiency around 2 and 100%, respectively, and was chosen to explore the TRPV1 mRNA expression in VSMCs.

**Table S1.** Nucleotide sequences of the custom designed primers for rat TRPV1; the primer that was used to examine the TRPV1 mRNA expression in primary rat aortic VSMCs is in bold.

| Primer set | Sequence |
| --- | --- |
| **Primer 1** | **fw: TTCACCGAATGGGCCTATGG**  **rev: TGACGGTTAGGGGTCTCACT** |
| Primer 2 | fw: GGGTGGACGAGGTAAACTGG  rev: TTCTCCCTGAAACTCGGCCT |
| Primer 6 | fw: CCTAGCTGGTTGCAAATTGGG  rev: TGGAGGTGGCTTGCAGTTAG |

***Extraction of total RNA from dorsal root ganglion***

Female Sprague Dawley rats of about 12 weeks of age were sacrificed. The spinal column was extracted and cut along the dorsal and ventral surfaces. The spinal cord was gently removed and single ganglia of lumbar and thoracical levels were harvested. The samples were then homogenised and lysed with TriFast, followed by chloroform extraction. RNA was precipitated using isopropanol and DEPC water.

***Calcium influx studies on HEK293T cells expressing the TRPV1***

Human TRPV1 was transiently expressed in HEK293T cells using jetPEI transfection reagent (Polyplus Transfection, Illkirch, France). Cells were loaded with Calcium 6 for 2 hours (Calcium 6 Kit; Molecular devices, San Jose, CA), in an extracellular solution containing (in mmol/L) 145 NaCl, 5 KCl, 10 glucose, 10 HEPES, 1.25 CaCl_2_, and 1 MgCl_2_, buffered to pH 7.4 with NaOH. Cells were loaded with the Calcium 6 reporter for 2 hours and then measured with a sampling frequency of 0.5 Hz, for a total of 150 seconds (Excitation 485 nm, Cutoff 515 nm, Emission 525 nm) using the FlexStation 3 (Molecular Devices, San Jose, CA). The antagonist (various concentrations of BCTC, as depicted) was pipetted automatically according to a preset protocol 20 seconds into the measurement. Capsaicin 0.5 μmol/L, was added 40 seconds thereafter. The area under the curve was calculated for each experimental run starting from the capsaicin application time point and used for computing the IC_50_.
