# Supplementary material for: Cultured rat aortic vascular smooth muscle cells do not express a functional TRPV1
Source: PLoS One. 2023 Feb 14;18(2):e0281191. doi: 10.1371/journal.pone.0281191 (PMC9928102; doi:10.1371/journal.pone.0281191)

Fig 1A. mouse mAb against C-termini of TRPV1 (Abcam) exp1

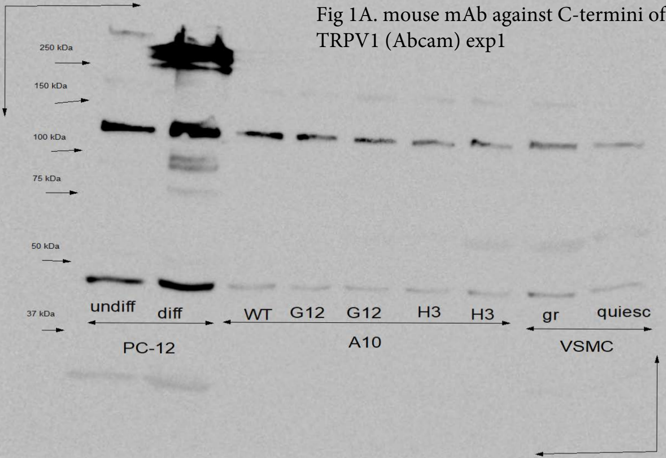

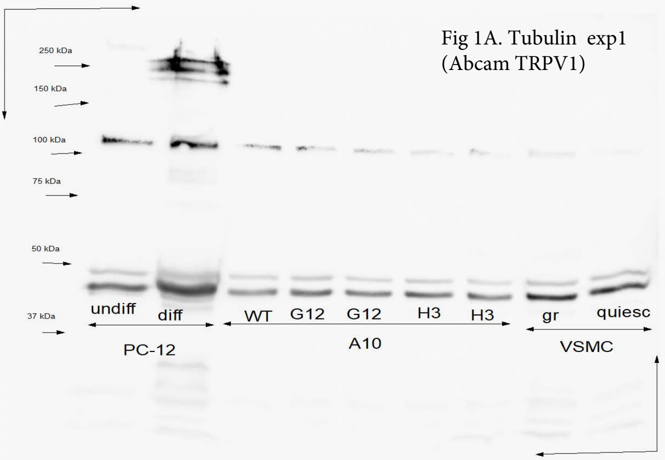
Fig 1A. Tubulin exp1
(Abcam TRPV1)
250 kDa
150 kDa
100 kDa
75 kDa
50 kDa
37 kDa
undiff
diff
WT
G12
G12
H3
H3
gr
quiesc
PC-12
A10
VSMC

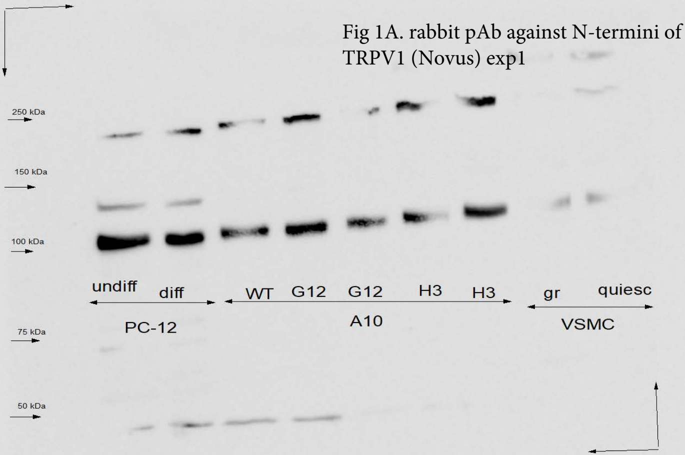
Fig 1A. rabbit pAb against N-termini of TRPV1 (Novus) exp1
250 kDa
150 kDa
100 kDa
75 kDa
50 kDa
undiff
diff
PC-12
WT
G12
G12
H3
H3
A10
gr
quiesc
VSMC

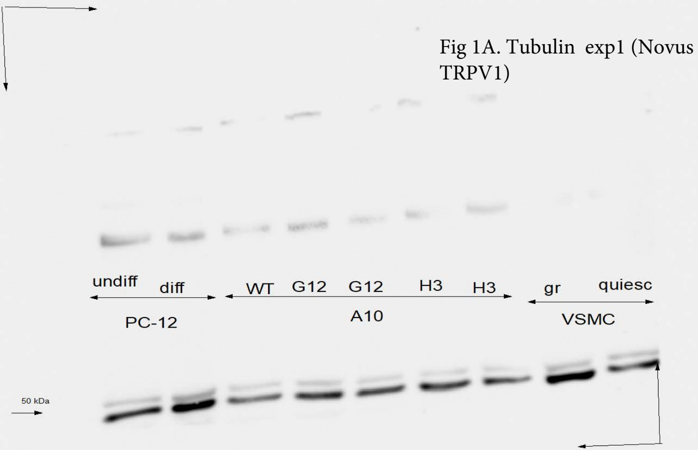
Fig 1A. Tubulin exp1 (Novus TRPV1)
undiff
diff
WT
G12
G12
H3
H3
gr
quiesc
PC-12
A10
VSMC
50 kDa

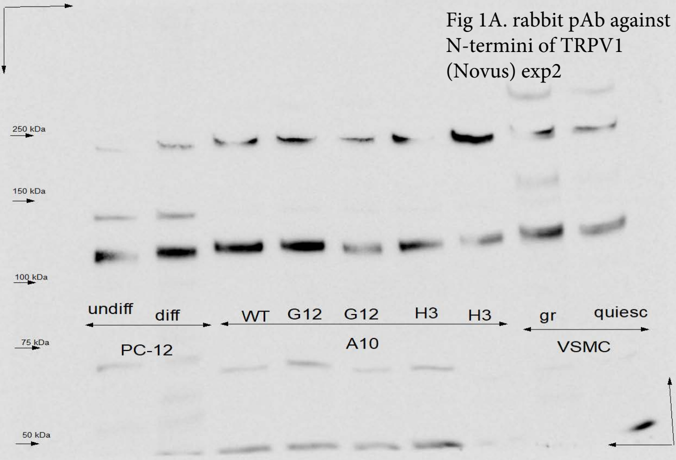

Fig 1A. rabbit pAb against N-termini of TRPV1 (Novus) exp2

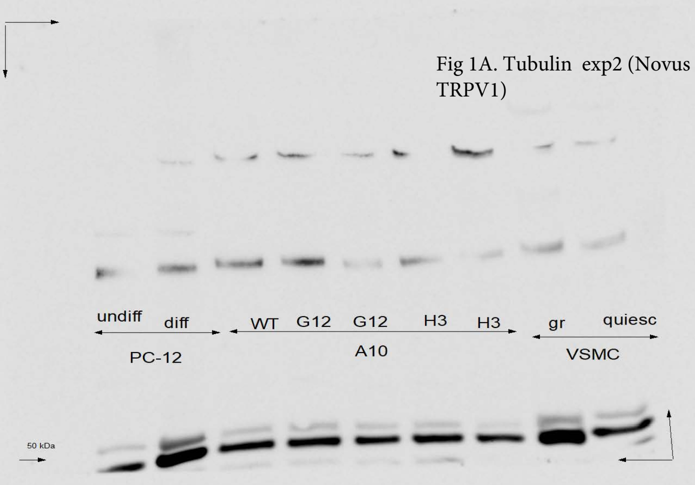
Fig 1A. Tubulin exp2 (Novus TRPV1)
undiff
diff
WT
G12
G12
H3
H3
gr
quiesc
PC-12
A10
VSMC
50 kDa

Fig 1A. mouse mAb against C-termini of TRPV1 (Abcam) exp2

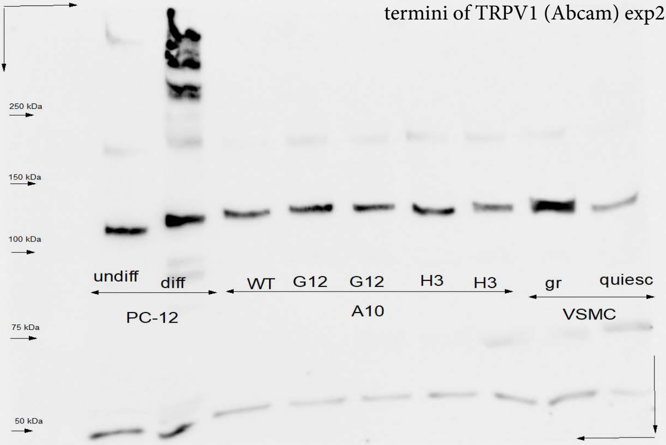

Fig 1A. Tubulin exp2 (Abcam TRPV1)

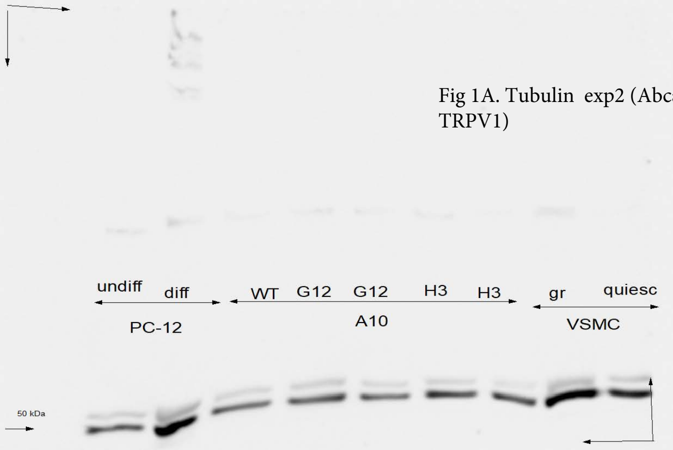

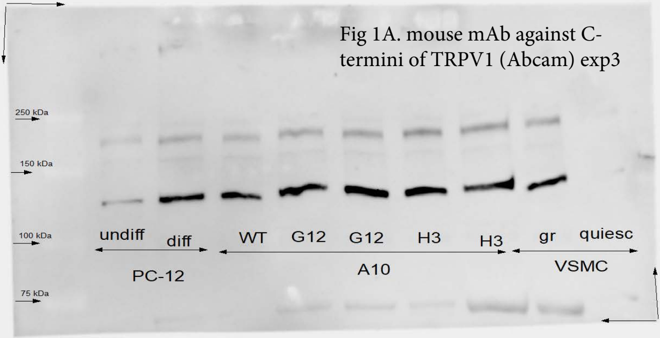
Fig 1A. mouse mAb against C-termini of TRPV1 (Abcam) exp3
250 kDa
150 kDa
100 kDa
75 kDa
undiff
diff
WT
G12
G12
H3
H3
gr
quiesc
PC-12
A10
VSMC

Fig 1A. Tubulin exp3
(Abcam TRPV1)

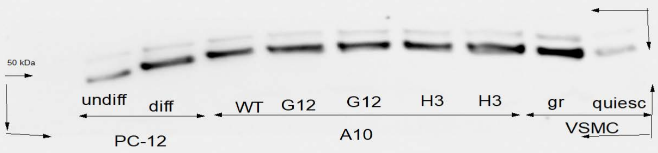

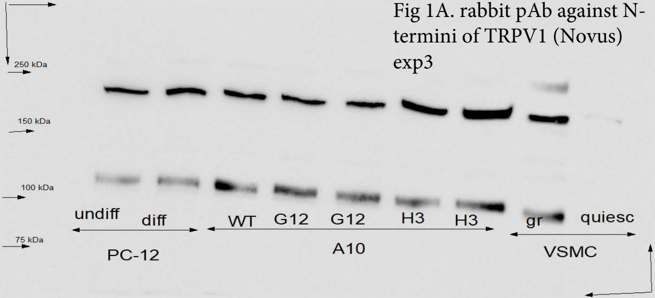

Fig 1A. rabbit pAb against N-termini of TRPV1 (Novus) exp3

Fig 1A. Tubulin exp3
(Novus TRPV1)

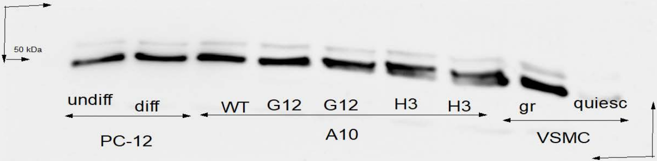

Fig 1B

pAb against N-termini ofTRPV1
(6% PAA gel) exp 1

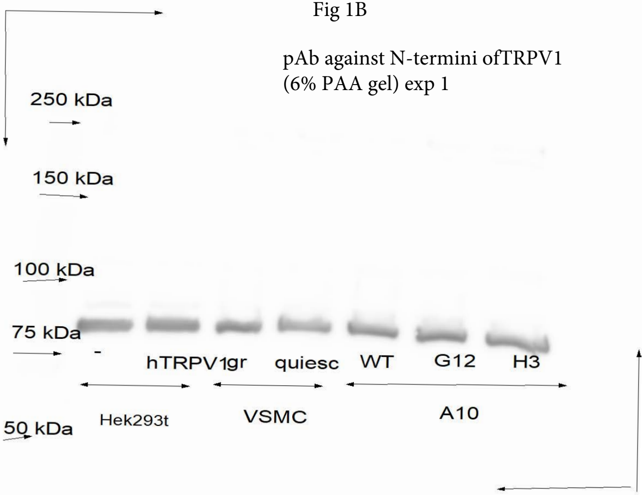

Fig 1B

tubulin (7.5% PAA gel) exp 1

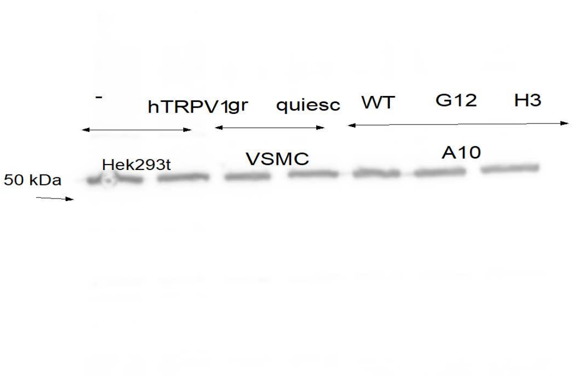

Fig 1B

pAb against N-termini ofTRPV1 (6% PAA gel) exp 2

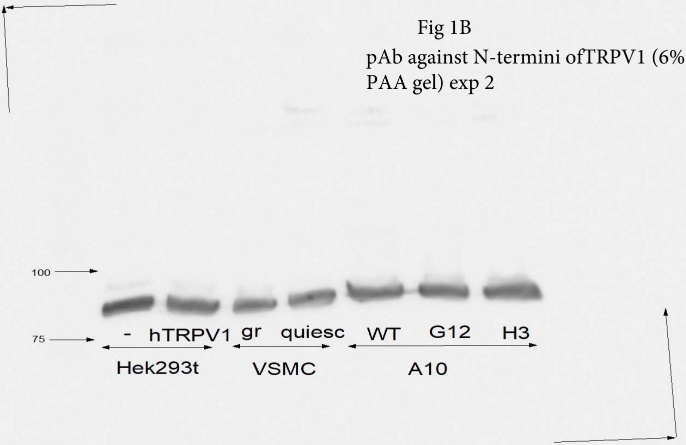

Fig 1B

tubulin (7.5% PAA gel) exp 2

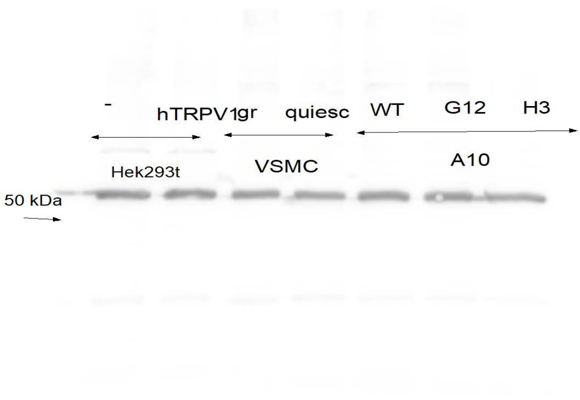

Fig 1B

pAb against N-termini ofTRPV1 (6% PAA gel) exp 3

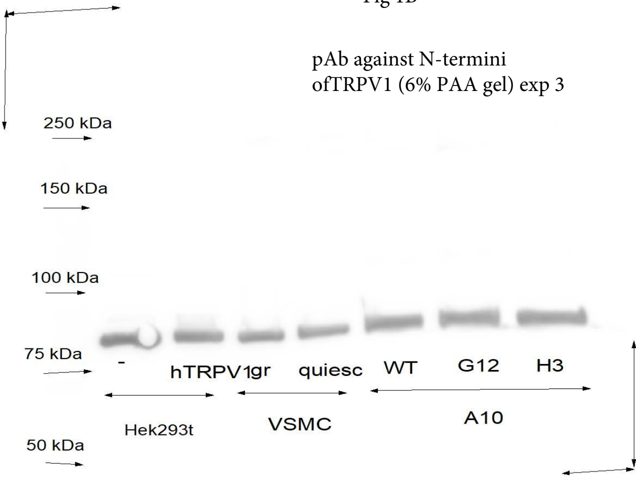

Fgi 1B

tubulin (7.5% PAA gel) exp 3

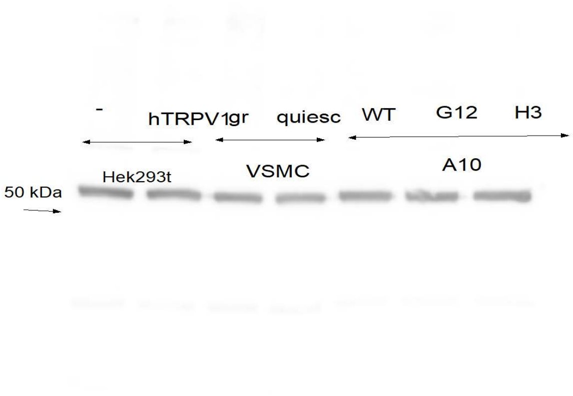

Fig 1B

pAb against N-termini ofTRPV1
(6% PAA gel) exp 4 - in the Fig 1B

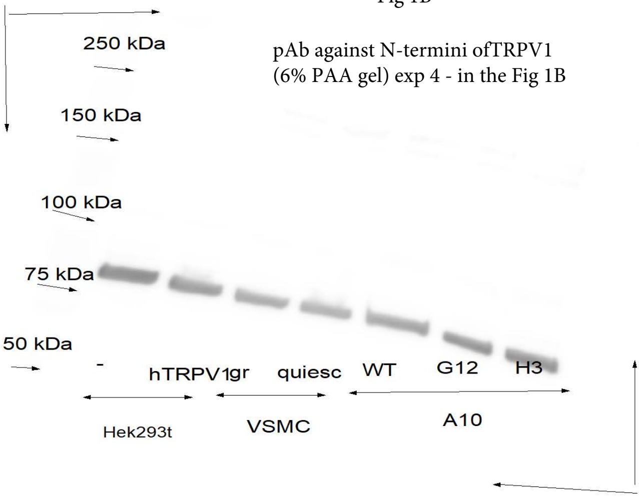

Fig 1B

tubulin (7.5% PAA gel) exp 4 - in the Fig 1B

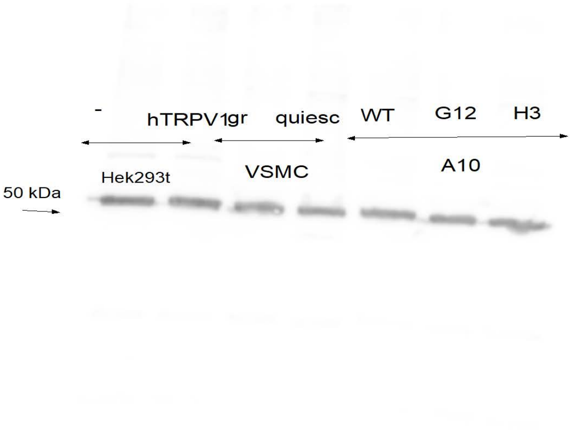

Supplement: S1 Raw images — (PDF) [file pone.0281191.s005.pdf]
